# Pediatric Palliative Care Pharmacy Pearls—A Focus on Pain and Sedation

**DOI:** 10.3390/children8100902

**Published:** 2021-10-10

**Authors:** Jennifer Placencia, Kevin Madden

**Affiliations:** 1 Department of Pharmacy, Texas Children’s Hospital, Houston, TX 77030, USA; 2 Department of Palliative, Rehabilitation and Integrative Medicine, University of Texas MD Anderson Cancer Center, Houston, TX 77070, USA; kmadden@mdanderson.org

**Keywords:** pediatrics, pharmacology, pain, opioids, palliative care

## Abstract

Determining the optimal dosing regimen for pediatric patients is a challenge due to the lack of dosing guidelines and studies. In addition, many developmental pharmacology changes that occur throughout childhood that have profound impacts on the absorption, distribution, metabolism, and elimination of medications are commonly used in palliative care. Adding to that complexity, certain medications have different effects in the pediatric patient compared to the adult patient. Being aware of the pharmacokinetic changes, impact on neurodevelopment and unique medication factors that are present in pediatric patients helps clinicians treat the pediatric palliative care patient in the best and safest way possible.

## 1. Introduction

Working in pediatric palliative care (PPC) is very rewarding but is not without its challenges, especially in the realm of pharmacotherapy. The treatment of pain in children is complex. The first challenge faced by those in pediatrics is the vast neurodevelopmental spectrum that leads to widely variable expressions of pain. Additionally, the lack of dosing guidelines, studies, and commercially available formulations make creating the optimal dosing regimen for pediatric patients a challenge, particularly when combined with the many developmental pharmacology changes that occur throughout childhood. In addition, certain medications have different effects on the pediatric patient compared to the adult patient. Specific examples will be covered in this article, including fentanyl-related chest wall rigidity, methadone prolongation of the corrected QT (QTc) interval, neurodevelopmental impairment from benzodiazepines, and tramadol effects on respiratory function. Being aware of these unique factors helps clinicians treat the PPC patient in the best and safest way.

## 2. Developmental Pharmacology

Large-scale, randomized clinical trials are rare, causing us to sometimes rely on case series or case reports. This leads to hesitance to use medications that have not been well studied due to lessons learned from past tragedies, such as birth defects from thalidomide, gray baby syndrome from chloramphenicol, and kernicterus from trimethoprim/sulfamethoxazole. As a result, it is important to be familiar with the concepts of developmental pharmacology and to be able to apply them to the medications commonly used in palliative care.

**Figure 1:**
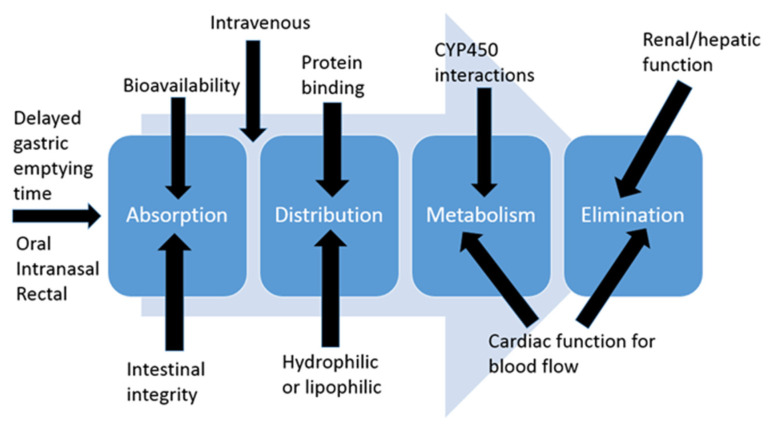
Absorption, distribution, metabolism, and elimination (ADME) overview.

Most clinicians that work with pediatrics have heard the statement “Children are not just small adults”. Further, it is important to remember that neonates are not just small children and premature neonates are not just small neonates. This is important to understand because most of the changes in developmental pharmacology occur in the first year of life, so neonates and infants will be the focus of this section. Developmental pharmacology concepts are broken down into absorption, distribution, metabolism, and elimination (ADME) categories (Figure 1).

### 2.1. Absorption

Intestinal Integrity: Premature neonates have immature intestinal integrity. Commercially available formulations of oral medications often contain high amounts of sugars, preservatives, and flavorings, which increases the osmolality of medications. In all age patients, these high osmolar medications can cause diarrhea, cramping, abdominal distention, and vomiting. In the youngest population, it can also increase their risk of developing necrotizing enterocolitis [1]. Therefore, these products are often avoided in premature neonates until they can tolerate a good amount of their feeds.

Intestinal Permeability: Neonates also have altered permeability of intestinal mucosa, which plays a role in medication absorption, notably increased absorption. Oral bioavailability (BA) of morphine is 19–47% (23.9%) in adults but 45–50% in term neonates [2]. As a result, neonates do not often need the 3–5 times dose when switching from IV to oral morphine. Doubling the dose will usually suffice in neonates.

Gastric Transit Time: Gastric emptying time is prolonged in the premature neonate, which can slow the absorption rate and onset of medication effect. As a clinical application of this, clinicians need to make sure to give oral medications enough time to work when using them for procedural pain or sedation.

### 2.2. Distribution

Volume of Distribution: Determining the dose needed to obtain certain plasma concentrations is the concept of volume of distribution. This is often affected by the fluid status in a patient. The percentage of total body water and extracellular water is highest in our premature neonatal population and decreases as they get larger such as term neonates and young infants [3]. As a result of having a higher percentage of their weight be water, the younger patients have a larger volume of distribution for hydrophilic medications, since those medications have farther to spread out in the body. Since they have a lower percentage of their weight as fat, they have a smaller volume of distribution for lipophilic medications such as lorazepam.

Protein Binding: Only the free (unbound) portion of a medication can exert its effects. Neonates not only have lower amounts of binding proteins but the proteins may have less affinity for medications [3]. The result of these two factors means there may be increased clinical effects of the same mg/kg dose of medication due to the increased free fraction of medication. For example, morphine has 33–37% protein binding in adults but only 18–22% of protein binding in neonates [4].

Blood–Brain Barrier (BBB): Neonates have an immature BBB, which can result in faster and greater medication uptake into the CNS. This can cause increased sensitivity to both the therapeutic and toxic effects of a medication. For example, typically lipophilic medications cross the BBB more easily than hydrophilic medications. Since morphine is a hydrophilic medication, it does not cross over to a great extent in older patients; however, it will cross over in neonates due to the immature BBB. This may be why neonates are more sensitive to the CNS effects of morphine.

### 2.3. Metabolism

Metabolism occurs in many sites in the body, but the major site of drug biotransformation is the liver. Neonates are born with much lower amounts of cytochrome p450 system enzymes compared to adults, which results in reduced clearance of some medications, especially in the first weeks of life [3]. Even though it starts off very low, these enzymes increase to over adult values by 1-year postnatal age, which is why dosing of some medications is more frequent in children than in adults. Fentanyl is primarily metabolized by CYP3A4 into inactive norfentanyl. Only a small amount is eliminated unchanged via the kidneys. Plasma clearance of fentanyl correlates with gestational age, and to a lesser degree body weight, and increases as patients get older and bigger [5]. As a result, lower doses are recommended for at least the first two weeks of life in all patients, especially the smallest and youngest of patients. Infants status post abdominal surgery also had lower clearance than expected, which suggests the increased abdominal pressure may limit fentanyl clearance [5]. Patients who are sicker often have a decrease in CYP3A4 activity. Neonates whose mothers received steroids before delivery might have higher CYP3A4 activity, since dexamethasone induces this enzyme [6].

Morphine, acetaminophen, lorazepam, and dexmedetomidine all undergo 3-glucuronidation. The activity of this process is reduced at birth and reaches close to adult levels by 2–6 months of age [4]. Almost all morphine is converted to the 3- and 6- glucuronide metabolites (M3G and M6G). M3G is an opioid antagonist, respiratory stimulant, and contributes to the development of tolerance. M6G is a potent opioid agonist and respiratory depressant. Preterm and young neonates produce mostly M3G at the beginning of life, so if not responding to morphine, conversion to another opioid is warranted [4]. Since lorazepam also relies on glucuronidation to get to an inactive metabolite, its half-life is much longer in younger patients. Neonates have a half-life of around 40 h compared to children at around 17 h and adults at 12 h. Therefore, the dosing interval must be extended in our younger patient population [7]. Infants also have reduced dexmedetomidine clearance due to impaired glucuronidation and CYP-450 enzyme activity. For patients between 30 and 60 weeks’ postmenstrual age (PMA), clearance increased by approximately 5% for every 1-week increase in PMA. Patients with recent cardiac surgery and on mechanical ventilation also showed lower clearance values, possibly due to lower cardiac output and blood flow to their organs [8].

Another pathway that is decreased in newborns is glycine conjugation, although it does increase to adult levels around 8 weeks of age. In the first two months, products that contain the preservative benzyl alcohol should be avoided if possible. Benzyl alcohol is oxidized to benzoic acid, conjugated with glycine, and excreted as hippuric acid. Since conjugation is reduced, benzoic acid accumulates, causing metabolic acidosis and possible neonatal gasping syndrome [9]. Some products do not have different options for preservatives, such as lorazepam, so caution must be used in neonates.

Unlike glucuronidation and glycine conjugation, the sulfotransferase system is well developed at birth such that it can compensate for the limited function of other pathways. For example, acetaminophen is mostly metabolized by glucuronidation in adults; however, neonates shift some of the metabolism to the sulfation pathway [10]. As a result, neonates have a longer acetaminophen half-life, but not as prolonged as it would be if it was only eliminated via glucuronidation.

### 2.4. Elimination

After a medication is metabolized, it needs to be eliminated from the body. The most common way is via the kidneys. Glomerular filtration rate (GFR) for full-term neonates at birth is approximately 20% of adult values and doubles around Week 2 of age. Premature neonates have an even lower GFR, since nephrogenesis is not complete until around 34 to 36 weeks’ gestation [11]. Serum creatinine is not an accurate marker of renal function in neonates as it is in adults because it reflects maternal creatinine for the first 1–2 weeks of life and because it is reabsorbed due to the neonate’s leaky tubules. The more premature a baby is born, the lower their renal function is, and the longer it takes them to reach adult values.

As a result, many medications have much longer half-lives in neonates. For example, the half-life of morphine can be as long as 10–12 h in preterm neonates yet decreases to 2–4 h in adults [4]. If dosing for a medication has not been studied in neonates and it is eliminated renally, caution should be used because of the likelihood of reduced elimination.

## 3. Unique Characteristics of Common Medications Used to Treat Pain in Children

### 3.1. Neurodevelopmental Impairment with Analgesics and Sedatives

Animal studies have demonstrated that brain development is negatively affected by benzodiazepines, and that is starting to be confirmed with neonatal studies [12]. Midazolam use in preterm infants causes hippocampal alterations, which can affect memory formation. Midazolam also binds to gamma-aminobutyric acid receptors, which are necessary for neuronal connection and communication in the neonatal brain. Without those receptors available, neuroapoptosis occurs [12]. This will have the most impact in the neonatal period, since that is a period of rapid brain development. More will be learned about this as studies are performed that follow these patients as they age, but benzodiazepines should be used judiciously in this population.

### 3.2. Fentanyl

A concerning side effect of fentanyl is chest wall rigidity. This is commonly observed in adult patients after administration of large doses and rapid infusions. However, in the Neonatal Intensive Care Unit (NICU), it has been seen with commonly administered doses for pain and sedation (3–6 mcg/kg) administered as a slow bolus over 2–3 min [13]. While the percentage of NICU patients in whom this occurs is low (around 5% of patients), it is something to be cautious about. Boluses should always be administered slowly, as should the flush that follows the bolus to clear the line.

### 3.3. Tramadol

Tramadol is a medication that is typically viewed as an opioid, albeit a “weak” mu-agonist. It is perceived to have the same potency as codeine and, as such, is often prescribed when health care providers want to avoid “stronger” opioids such as morphine, oxycodone, or hydromorphone. Pharmacological data suggest that the affinity of tramadol for the mu-receptor is approximately 10 times less than codeine and 6000 times less than morphine [14]. The affinity of tramadol for the delta and kappa opioid receptors is even less. Given this, it is difficult to attribute the pain-relieving qualities of tramadol solely to activation of the mu-receptor.

So how does tramadol relieve pain? Essentially tramadol works in two ways. The first is through an active metabolite of tramadol, O-desmethyltramadol. Tramadol is metabolized to O-desmethyltramadol via the CYP2D6 cytochrome P450 enzyme system, and O-desmethyltramadol binds to mu-opioid receptors.

The second way tramadol relieves pain is by blocking the reuptake of serotonin and norepinephrine, much duloxetine and venlafaxine, which are serotonin-norepinephrine reuptake inhibitors (SNRIs). Both duloxetine and venlafaxine are used for neuropathic pain, and there is some evidence that they may also have an anti-inflammatory effect.

In addition to its unique mechanisms of action, tramadol also has some safety concerns in the pediatric population. As mentioned above, tramadol is metabolized by CYP2D6 into its active metabolite. There is variable expression of CYP2D6 across humans. Some people are ultrarapid metabolizers, some are poor metabolizers, and many have normal metabolism. Ultrarapid CYP2D6 metabolizers are individuals who have more than two copies of functional CYP2D6 alleles. In these patients, a higher percentage of tramadol is metabolized to active metabolites, causing a greater risk of respiratory depression and/or death.

From 1969 to 2016, the Federal Drug Administration (FDA) discovered nine cases of respiratory depression in children less than 18 years of age, three of them involving deaths. As a result, they released warnings and contraindications to the use of tramadol in pediatric patients [15]. Contraindications include all children younger than 12 years and children younger than 18 years who are receiving it to treat pain after surgery to remove tonsils and/or adenoids. There was also a warning about use in adolescents between 12 and 18 years who are obese, have obstructive sleep apnea (OSA), or have severe lung disease (SLD).

Commonly known side effects of tramadol include seizures, hypoglycemia, and serotonin syndrome. Interestingly, despite its low affinity for opioid receptors, tramadol has a somewhat higher risk of prolonged use compared with other short-acting opioids.

The potential benefits of tramadol must be weighed against its side effect profile before administering.

### 3.4. Gabapentin

Gabapentin was initially developed in the 1970s to treat spasticity. It was approved by the United States Federal and Drug Administration in the 1990s to treat postherpetic neuralgia. Yet, there are thousands of citations on PubMed for the treatment of pain with gabapentin. Gabapentin is extensively used in PPC, and children with severe neurologic injury often utilize these services. Gabapentin has been shown to be helpful for neuropathic pain and “neuroirritability”.

Gabapentinoids (gabapentin and pregabalin) function by binding to presynaptic neuronal alpha-2-delta subunits of voltage-gated calcium channels. This leads to downregulation and decreased production of substance P, glutamate, and calcitonin-related gene peptide, which are thought to initiate and maintain neuropathic pain states.

One of the more common side effects of gabapentinoids is somnolence. The drowsiness associated with gabapentinoids is thought of as occurring in the initial days of treatment, after which the patient becomes accustomed to the drug and somnolence improves. Due to case reports of prolonged hypoventilation in postoperative patients receiving gabapentinoids [16], the product monograph was amended to include a warning that respiratory depression may occur when given simultaneously with opioids. Recent population-based studies report found an association between the coprescription of opioids and gabapentinoids and an increased risk of opioid-related death [17,18]. Patients with advanced cancer and/or receiving palliative care, however, were excluded from this study.

There is limited evidence to support the use of gabapentinoids for cancer-related neuropathic pain due to the low quality of published data and a lack of efficacy when compared to opioids alone. In fact, the clinical practice guideline of the American Society of Clinical Oncology recommends the use of duloxetine over tricyclic antidepressants and gabapentinoids. Given the potential risk of unintentional overdose when gabapentinoids are used with opioids, it may be prudent for palliative care physicians to consider alternative medications for the first-line treatment of neuropathic pain in patients with cancer who also are taking opioids. For children 7 years of age or older, duloxetine is equally as efficacious as gabapentin without the untoward side effect profile.

### 3.5. Methadone

Methadone is a unique opioid in that it modulates pain via multiple receptors. Methadone provides agonist activity at the μ-, δ-, and κ-opioid receptors, antagonism at the N-methyl-D-aspartate receptor, and is also a serotonin-norepinephrine reuptake inhibitor. In addition to its high bioavailability, long half-life, and no active metabolites, it is particularly attractive to those in pediatrics, as it is the only long-acting opioid that comes as a liquid. This allows even the youngest patient, or one with impaired swallowing and can only receive medications through a nasogastric or gastric tube, to access the benefits of long-acting opioids.

Unfortunately, there is a great amount of trepidation in prescribing this opioid. The long half-life leads many to believe it is a difficult medication to titrate—patience, however, is often rewarded with superior analgesia than other pure mu-agonists, especially in those with mixed nociceptive and neuropathic pain. Perhaps the biggest barrier to prescribing methadone is its fabled association with a prolonged corrected QT interval (QTc) that can lead to Torsade de Pointes, a fatal cardiac arrhythmia. There are generally two misconceptions that perpetuate the concern with methadone: (1) what constitutes a “normal” QTc interval and (2) the frequency of a prolonged QTc interval.

Most pediatric health care providers define a “prolonged” QTc interval as greater than 450 milliseconds (ms) [19]; data suggest, however, that the normal 99–99.5th percentile values are 460 ms for prepubertal children, 470 ms for pubertal males, and 480 ms for pubertal females [20]. The importance of these values cannot be understated. On the one hand, the decision to not use methadone based on incorrect normative QTc values can cause great suffering to a child who may benefit from this exceptional opioid, while on the other hand, the decision to prematurely stop methadone in a child whose QTc is, for example, 460 ms may be just as detrimental.

In general, methadone is exceedingly well tolerated in children, with little evidence of clinically significant prolongation of the QTc interval. Caution, however, is warranted when using in critically ill children, those on azole antifungal medications, or with electrolyte abnormalities. In these children, vigilance with frequent ECG monitoring is recommended.

## 4. Opioid-Induced Hyperalgesia

Often, we escalate doses of opioids when a patient appears uncomfortable. This might be the correct path if tolerance or untreated pain is present; however, keep in mind the potential for opioid-induced hyperalgesia (OIH).

Hyperalgesia is hypothesized to occur via many mechanisms; one main contributor may be glutamate-associated activation of NMDA receptors, which causes an augmented excitatory response [21]. Risk factors for hyperalgesia include high doses of opioids, rapidly escalated opioid doses, parenteral administration of opioids, use of short-acting opioids compared to longer-acting ones, and receiving a phenanthrene opioid (such as morphine, hydromorphone, or oxycodone) [21,22,23].

With hyperalgesia, the pain quality, location, and distribution pattern are likely to be different from their original pain condition. The pain is usually diffuse, has neuroexcitatory effects, and may be accompanied by myoclonus, hyperesthesia, or allodynia [22]. Management of OIH is dose reduction and/or addition of adjunctive agents [23].

If OIH is suspected, the dose of the opioid should be reduced; however, it is usually a challenge to get patients/caregivers to agree with this because the patient is often experiencing their highest peak of pain. Clinicians should always have a backup plan in place in case the pain worsens.

Codeine, hydromorphone, morphine, and other structurally similar opioids in the phenanthrene class undergo glucuronidation to a NMDA receptor-activating metabolite. Since NMDA activation promotes hyperalgesia, switching to an opioid that is structurally unique (outside of the phenanthrene class like fentanyl) could reduce the hyperalgesia [24,25].

The use of adjunctive agents should also be considered in order to block the activation of the NMDA receptor to reduce the spinal neuron sensitization. Methadone and ketamine can block NMDA receptors and provide pain relief via different receptors than the opioid from which the patient is experiencing OIH. Adding an adjunctive agent, even outside of NMDA antagonism such as acetaminophen or ketorolac, should be considered for the goal of being able to administer lower opioid doses.

## 5. Conclusions

When one considers the aspects of developmental pharmacology as well as unique metabolic patterns and the lack of clinical studies regarding medications that can be commonly used to treat pain in children, the treatment of pain can almost feel overwhelming. As with most things in medicine, start with the initial conservative dose (never be ashamed to consult your pharmacopeia of choice), assess the response, observe for side effects, and titrate accordingly!
